# A mouse model and pathogenesis study for CVA19 first isolated from hand, foot, and mouth disease

**DOI:** 10.1080/22221751.2023.2177084

**Published:** 2023-02-15

**Authors:** Wangquan Ji, Ling Tao, Dong Li, Peiyu Zhu, Yuexia Wang, Yu Zhang, Liang Zhang, Shuaiyin Chen, Haiyan Yang, Yuefei Jin, Guangcai Duan

**Affiliations:** a Department of Epidemiology, College of Public Health, Zhengzhou University, Zhengzhou, People’s Republic of China; b Academy of Medical Science, Zhengzhou University, Zhengzhou, People’s Republic of China; c School of Public Health, Xinxiang Medical University, Xinxiang, People’s Republic of China

**Keywords:** Coxsackievirus A19, hand, foot, and mouth disease, oral infection, diarrhoea, encephalomyelitis

## Abstract

*Coxsackievirus* A19 (CVA19) is a member of Enterovirus (EV) C group in the *Picornaviridae* family. Recently, we reported a case of CVA19-infected hand, foot, and mouth disease (HFMD) for the first time. However, the current body of knowledge on the CVA19 infection, particularly the pathogenesis of encephalomyelitis and diarrhoea is still very limited, due to the lack of suitable animal models. Here, we successfully established a CVA19 mouse model via oral route based on 7-day-old ICR mice. Our results found the virus strain could directly infect the neurons, astrocytes of brain, and motor neurons of spinal cord causing neurological complications, such as acute flaccid paralysis. Importantly, viruses isolated from the spinal cords of infected mice caused severe illness in suckling mice, fulfilling Koch’s postulates to some extent. CVA19 infection led to diarrhoea with typical pathological features of shortened intestinal villi, increased number of secretory cells and apoptotic intestinal cells, and inflammatory cell infiltration. Much higher concentrations of serum cytokines and more peripheral blood inflammatory cells in CVA19-infected mice indicated a systematic inflammatory response induced by CVA19 infection. Finally, we found ribavirin and CVA19 VP1 monoclonal antibody could not prevent the disease progression, but higher concentrations of antisera and interferon alpha 2 (IFN-α2) could provide protective effects against CVA19. In conclusion, this study shows that a natural mouse-adapted CVA19 strain leads to diarrhoea and encephalomyelitis in a mouse model via oral infection, which provides a useful tool for studying CVA19 pathogenesis and evaluating the efficacy of vaccines and antivirals.

## Introduction

Human *Enteroviruses* (HEVs) are common pathogens causing systemic manifestations, belonging to the family of *Picornaviridae* that includes four enterovirus species (i.e. HEV-A, HEV-B, HEV-C, and HEV-D). HEVs infections are typically asymptomatic or mild, but serious complications occur in some patients, especially in infants and individuals with weakened immune systems, emphasizing their continuing threat to public health [1]. Among HEVs, *Coxsackieviruses* are associated with a diverse spectrum of diseases, including hand, foot, and mouth disease (HFMD), aseptic meningitis (AM), acute flaccid paralysis (AFP), acute flaccid myelitis (AFM), conjunctivitis, encephalitis, myocarditis, respiratory problems, and diarrhoea [2,3]. *Coxsackievirus* A19 (CVA19) is a positive-sense, single stranded RNA virus classified under the species of the HEV-C group. Multiple studies have reported that CVA19 causes acute gastroenteritis in many countries, such as India [4], Thailand [5,6], Japan [7], and France [8]. However, like other neurotropic HEVs, CVA19 can also result in AFP [9], AM [10], demonstrating the ability to directly infect the central nervous system (CNS). Recently, we reported a HFMD case infected by CVA19 for the first time [11], indicating CVA19 became an emerging pathogen of HFMD. Because of continuous evolution of HEVs, some emerging HFMD-associated pathogens have expanded the spectrum of pathogens and symptoms among HFMD patients. For example, tomato flu reported in India is considered to be a new variant of the HFMD, which is associated with a novel variant strain of CVA16 [12]. Given the neurotropic ability of CVA19, the outbreak of CVA19 may cause more serious consequences, which should be taken seriously. Unfortunately, prophylactic and therapeutic agents against CVA19 infection have not been well developed so far. Animal models have provided an indispensable tool in understanding human diseases. Therefore, the development of an appropriate animal model will greatly contribute to CVA19 pathogenesis, and subsequently provides valuable information on the treatments and prevention for the disease.

To establish an animal model, the main optimization factors are virus strains, species of mice, infection route, inoculation dose, and age of mice. Several mouse models of HFMD have been established, but the main infection routes are intraperitoneal (i.p.), intramuscular (i.m.), and intracranial (i.c.) [13]. Nevertheless, since those routes are not the natural infection route, the application will be limited if we establish the CVA19 mouse model imitating these infection routes. Because of species specificity, HEVs are extremely difficult to naturally cause disease in mice through oral route. Researchers have tried to address this problem by using transgenic mice [14], immunodeficiency mouse [15], and mouse-adapted strains [16] or the combination of the above approaches. Transgenic mice are of producing high cost, and the immunodeficient mice hardly reflect the real infection due to defective immune systems [13]. In this study, we isolated an emerging CVA19 strain from a HFMD patient, naturally adapted to mice, and successfully established an orally infected CVA19 mouse model based on 7-day-old ICR mice, which manifested diarrhoea and encephalomyelitis similar to human infection. Taken together, our study provides a mouse model that mimics the natural route of infection for studying CVA19 pathogenesis and evaluating the efficacy of vaccines or antivirals.

## Materials and methods

### Ethics statement

The ICR mice were purchased from Beijing Vital River Laboratory Animal Technology Co., Ltd. and housed under specific-pathogen-free conditions in ventilated cages (IVC, Tecniplast, Milan, Italy) of Animal Center in College of Public Health of Zhengzhou University on a 12 h light/dark cycle with ad libitum access to food and water. All animal experiments were strictly performed according to the guidelines of laboratory animal ethics. And experimental protocols were approved by the Life Science Ethics Review Committee of Zhengzhou University (permission no: ZZUIRB2020-29).

### Preparation of viral stocks

The original CVA19 strain (GenBank: MT175706.1) was isolated from the stool sample of a child with HFMD [11]. It is worth noting that this CVA19 strain is unable to proliferate on cultured RD cells and Vero cells in *vitro*, but naturally adapted to mice. To prepare viral stocks, 3-day-old mice were i.p. inoculated with CVA19. When infected mice exhibited severe signs of limb paralysis, skeletal muscles of paralysed mice were dissected and grinded (10% wt/vol) with PBS in a sterile condition, and then centrifuged at 12000×g for 10 min at 4°C. To obtain control muscle homogenate supernatant, the skeletal muscles of normal mice were dissected and prepared as above. Subsequently, the supernatant was separated and filtrated with a 0.22 μm filter, and then aliquoted and stored at −80°C [16]. The virus titre of filtered supernatant of tissue homogenates was 1.47 × 10^10^ viral RNA copies/μL.

### Animal infection experiments

All mice were fasted for 6 h before inoculation. To optimize the appropriate dose of challenge, 7-day-old ICR mice were infected with 10^4^, 10^6^, 10^8^, 10^10^ copies/mouse (*n* = 10 per group) via intragastric (i.g.) route applying self-made needle. To explore the reasonable age, three groups of 5-, 7- and 10-day-old ICR mice (*n* = 7 or 8 per group) were i.g. inoculated with 10^8^ copies/mouse. Control mice were inoculated with an equal volume of muscle homogenate supernatant from normal mice and kept in a separate cage. Animals were observed daily for clinical signs of back hunching, ruffled fur, weight loss, and limb paralysis. Clinical scores were graded as follows: 0, healthy; 1, lethargy and inactivity; 2, ataxic; 3, weight loss; 4, limb paralysis; 5, dying or death.

### Viral loads

Seven-day-old ICR mice were i.g. inoculated with CVA19 (10^8^ copies/mouse) using a self-made needle. Animals were closely observed for clinical signs, and sequentially euthanized at various time points. Tissue samples were harvested, weighed, and stored at −80°C. Total RNA was extracted by using 1 mL TRIzol reagent (Thermo Fisher Scientific Inc., MA, USA) on the basis of manufacturer’s methods. Total RNA of blood samples was extracted using an RNAiso Blood kit according to the manufacturer's instructions (Takara Bio Inc., Code No. 9112Q). One microgram of total RNA was reverse transcribed into cDNA using a kit (Yeasen Biotechnology (Shanghai) Co., Ltd., Shanghai, China) according to the manufacturer’s instructions. The genomic RNA of CVA19 was measured by quantitative PCR (Yeasen Biotechnology (Shanghai) Co., Ltd., Shanghai, China) with a pair of primers, CVA19 VP1-R (CACACAAGCGGATCTCCCAA) and CVA19 VP1-F (AACTGGAGCAACTAGCCAGG), using the instrument (Kubo Tech Co., Ltd., Beijing, China) according to standard protocol. The target product (118 bp) amplified by the primers was inserted into the pET-28a (+) plasmid, producing the plasmid pET-28a (+)-CVA19 VP1, which was used as a standard for absolute quantification of CVA19 copy numbers. The standard curve was generated from serially diluted pET-28a (+)-CVA19 VP1 (ranging from 10^2^ to 10^11^ copies/μL) by linear regression. Viral loads were calculated with a standard curve and were expressed as Log_10_ (viral RNA copies/mg tissues or mL blood).

### Histopathology

At 10 days post infection (dpi), animals with severe or critical illness were euthanized, and the harvested tissue samples were fixed by immersion in 4% paraformaldehyde. The tissue samples were bisected, embedded in paraffin, and stained with haematoxylin and eosin (H&E), Nissl’s stain, Periodic acid Schiff reagent (PAS) glucogen stain, Alcin blue (AB) stain. To evaluate the degree of inflammation in intestinal samples, a modified scoring system [17] was used by two pathologists blinded to treatment conditions. Five factors need to be considered to give a comprehensive pathological score, including villous atrophy, epithelial injury, acute inflammation, chronic inflammation, and depth of inflammation, on a scale of 0∼3, thus yielding an additive score between 0 (no mucositis) and 15 (maximal mucositis). The mean number of PAS, AB, and Cleaved-Caspase3, CD45, CD11b, and Ly6G positive cells in intestine slices were counted in at least 100 villi in each 1-cm long segment [18].

### Immunofluorescence staining (IF)

Paraffin sections were deparaffinized and rehydrated by incubating in xylene and dehydrating using graded ethanol. After retrieving antigen, the sections were blocked by 3% bovine serum albumin (BSA) for 30 min. Then, we threw away the blocking solution and incubated slices with primary antibody overnight at 4°C. Slices were washed three times with PBS (pH 7.4) and then covered with secondary antibody, incubating at room temperature for 50 min in dark condition. Subsequently, DAPI solution was dropped to counterstain the nuclei at room temperature for 10 min. Before covering slips with anti-fade mounting medium, the sections were added spontaneous fluorescence quenching reagent and incubated for 5 min. Microscopy detection and collect images by Fluorescent Microscopy (OLYMPUS, IX73).

### Fluorescent in-situ hybridization (FISH)

The in-situ hybridization probes and kits were designed and synthesized by Wuhan Servicebio Technology Co., Ltd. The digoxigenin (DIG)-labeled, CVA19-specific DNA probe sequences are as follows: 5‘-CCAATTATAGCCACACAAGCGGATCTCCCAAA-3’. Briefly, we added proteinase K working solution to each section and followed incubation with 3% methanol-H_2_O_2_ for 15 min; then added pre-hybridization solution and incubate for 1 h at 37°C, followed by adding the CVA19-specific DNA probe hybridization solution hybridizing overnight. After washing, the slices were incubated with a blocking solution for 30 min. Next, the mouse anti-DIG-HRP and TSA developing were added and the primary antibody was incubated at 4°C overnight. After washing, the section was incubated for 50 min with a secondary antibody, and then incubated with DAPI for 8 min. Finally, microscopic examination and photography were collected.

### Measurement of myeloperoxidase (MPO) activity

The MPO activity of small intestine removed from infected mice (score = 4 or 5) and control mice (*n* = 7 per group) was measured using a corresponding kit (Nanjing Jiancheng Bioengineering Institute), according to the manufacturer’s directions. The activity was expressed as units (U) per gram of wet weight (U/g).

### Transmission electron microscopy (TEM)

As previously described [19], the spinal cords of infected mice (*n* = 4) were harvested and immersed in special electron microscope fixator for at least 2 h. After washing with PBS three times, the final trimmed samples were post-fixed with 1% osmium tetroxide for 2 h. After dehydrating and applying a graded ethanol series, the specimens were embedded in Araldite 812. Finally, the ultrathin sections (90 nm) stained with uranyl acetate and lead citrate were examined by using a Hitachi HT7800 electron microscope at a voltage of 120 kV.

### Routine blood tests and cytokines

Mock- and CVA19-infected mice were sequentially euthanized at 3, 5, and 8 dpi (*n* = 4∼7 for each time point). Blood samples were collected by cardiac puncture. A small portion of fresh blood samples were subjected to routine blood tests applying automatic animal blood cell analyzer (Shenzhen Mindray Bio-medical Electronics Co. Ltd), and the rest of blood samples were used to separate serum to detect the concentrations of cytokines (IL-6, TNF-α, CXCL-1, and MCP-1) by Enzyme-linked immunosorbent assay (ELISA) (Biolegend, CA, USA).

### Western blotting (WB)

At 5 and 8 dpi, 3 mice with mild symptoms (clinical score up to 3) and 3 mice with severe symptoms (clinical score up to 4 and above) were euthanized respectively. The brains and spinal cords from control (*n* = 3) and infected mice were homogenized in RIPA buffer (Beyotime Institute of Biotechnology, Shanghai, China) with protease and phosphatase inhibitors to extract total proteins. After mixing with equal amount of 2× SDS loading buffer, equal amounts of protein were subjected to 10% SDS-PAGE and then transferred to PVDF membranes. Membranes were incubated with 5% skim milk powder in PBS at room temperature for 1 h. Subsequently, membranes were incubated with primary antibodies (1:1000 dilution) overnight at 4°C. Next, membranes were washed thrice with PBST and incubated with secondary antibodies (1:3000 dilution). Finally, membranes were washed thrice and developed with an enhanced chemiluminescence kit (Biosharp, Hefei, China). The integrated intensity of protein bands was calculated using Image J software.

### Antibodies

The following commercially available antibodies were used in this study: Rabbit anti-Cleaved Caspase-3 (Asp175) (5A1E) antibody (Cell Signaling Technology, Inc.; Cat#9664S), mouse anti-GFAP antibody (absin Bioscience Inc.; Cat# abs100080), mouse anti-β-actin (8H10D10) antibody (Cell Signaling Technology, Inc.; Cat# 3700S), rabbit anti-NeuN antibody (Wuhan Servicebio Technology Co., Ltd.; Cat# GB11138), rabbit anti-ChAT antibody (Wuhan Servicebio Technology Co., Ltd.; Cat# GB11070-1), rabbit anti-CD45 antibody (Wuhan Servicebio Technology Co., Ltd.; Cat# GB113886), rabbit anti-CD11b antibody (Wuhan Servicebio Technology Co., Ltd.; Cat# GB11058), and rabbit anti-Ly6G antibody (Wuhan Servicebio Technology Co., Ltd.; Cat# GB11229).

### Protective efficacy of CVA19-VP1 monoclonal antibody and ribavirin

To confirm the protective efficacy of the CVA19-VP1 monoclonal antibody (mAb) and ribavirin against CVA19 challenge *in vivo*, four lethal viral challenge experiments were carried out. Seven-day-old ICR mice were i.g. inoculated with CVA19 (*n* = 8–10 per group), followed by the i.p. administration of 10-fold and 100-fold diluted CVA19-VP1 mAb, and ribavirin (100 μg per mouse) (Shanghai Macklin Biochemical Co., Ltd., Shanghai, China) [20] within 1 h post-inoculation (hpi). Mock-infected mice were i.p. administrated with PBS. The mortality and clinical manifestation were monitored and recorded daily until 15 dpi.

### Protective effect of CVA19 antisera

To examine the protective effect of passive immunization, CVA19 antisera was collected from 4 survived CVA19-infected mice at 30 dpi. Negative serum was obtained from 4 mock-infected mice at 30 dpi. To test the neutralizing effect of antisera, seven-day-old ICR mice were i.g. inoculated with CVA19 (*n* = 8–13 per group), followed by the i.p. administration of 50 μL of original, 1:4, 1:16, 1:64, 1:128 diluted antisera or negative serum at 1 hpi. All mice were monitored daily for clinical symptoms or death until 15 dpi.

To evaluate the antiviral effects of type I IFNs, seven-day-old ICR mice were i.g. inoculated with CVA19 (*n* = 8–10 per group), followed by the i.m. administration of IFN-α2 (666.75 U/mouse) [20] or IFN-β (3 × 10^4^U/mouse) [21] (Sino Biological, Inc., Beijing, China). The mortality rates and clinical symptoms were then monitored and recorded daily until 15 dpi.

### Experiment to prove Koch’s postulates

Three indispensable postulates need to be fulfilled to demonstrate one infectious nature of the disease. The third hypothesis says: the agent has to be isolated from the patient and should, after further passages in pure culture, again cause this disease. Since CVA19 is difficult to be cultivated in multiple cell lines [22], we have to directly infect mice with lysates. To determine whether CVA19 was the direct cause of the paralytic disease in mice by fulfilling Koch’s postulates [23], the spinal cord lysates from mice with mild symptoms (clinical score up to 3) and severe symptoms (clinical score up to 4 and above) were removed respectively and then homogenized in PBS (10% wt/vol). The spinal cord lysates were filtered by a 0.22-μM filter, and then infected two groups of 3-day-old ICR mice via i.p. route (*n* = 8 per group), respectively. Control mice (*n* = 8) were administrated with negative spinal cord lysates from healthy mice. The mortality and clinical manifestation were monitored and recorded daily until 7 dpi.

### Statistical analysis

GraphPad Prism 8 software (GraphPad 8.3 Software, San Diego, CA, USA) was employed to statistically analyse the difference in survival rate, weight, mean clinical score between different groups. The differences of survival rates were assessed with the Mantel–Cox Log-rank test and survival curves were plotted using the Kaplan-Meier method. Furthermore, the results were expressed as the mean ± standard deviation (SD) or median with range (non-normal distribution). The variance analysis was conducted by two-tailed Student’s *t-test* or Mann-Whitney test (non-normal distribution) on performing the comparison of two groups. *P *< 0.05 indicated statistical significance. All experiments were repeated at least three times.

## Results

### The CVA19 strain is naturally adapted to mice and induces limb paralysis in neonatal mice with an age- and dose-dependent manner

**Figure 1.**
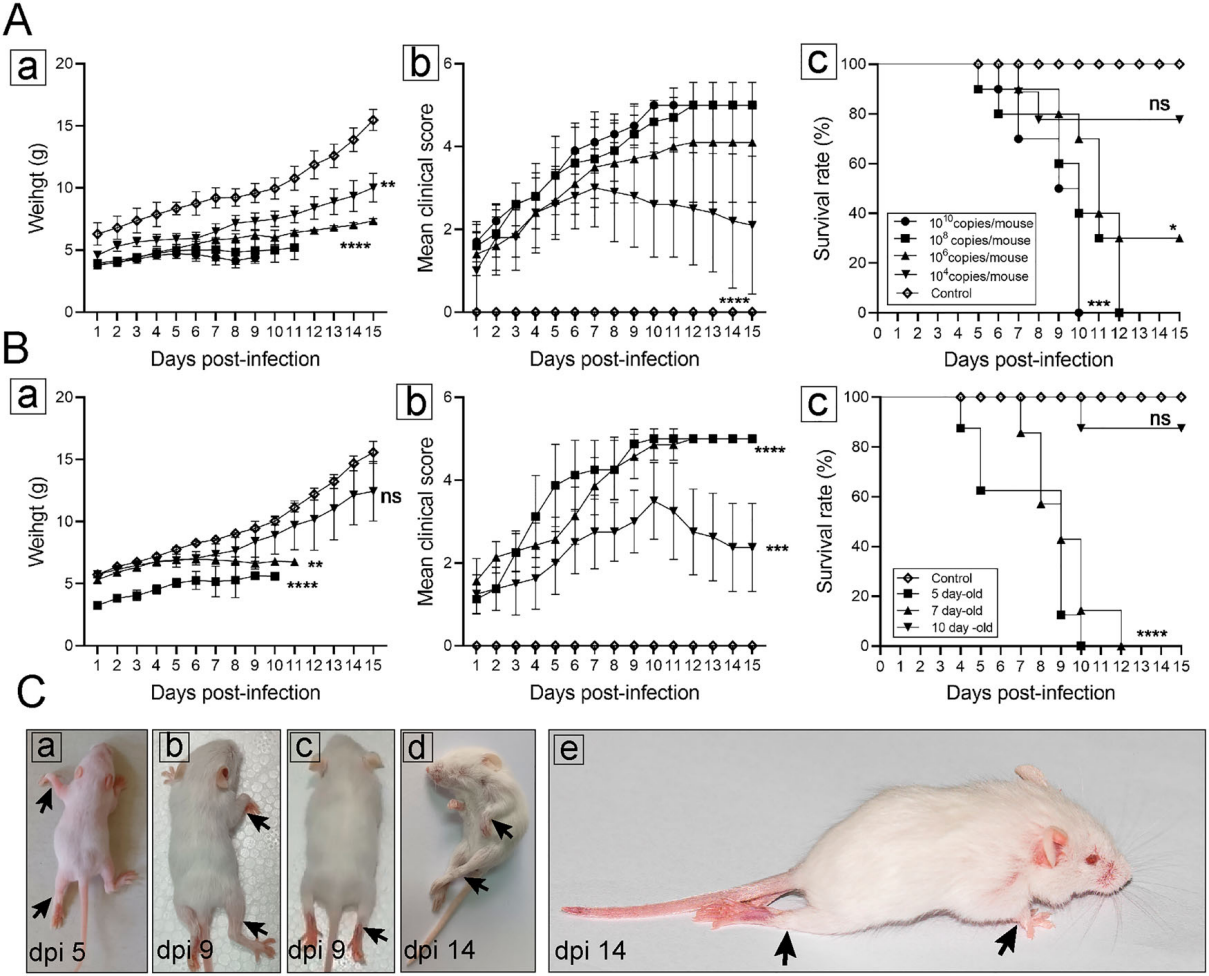
Establishment of CVA19 mice model. The body weight (A-a and B-a), clinical scores (A-b and B-b), and survival rates (A-c and B-c) were monitored and recorded daily until 15 dpi, in the inoculation dosage-, and age-dependent experiments. (C) Five representative pictures of clinical signs. The black arrows indicated the paralysed limbs of infected mice. Data represent the mean ± SD. Different groups *vs* control, *P < 0.05, **P < 0.01, ***P < 0.001, ****P < 0.0001; ns, non-significant.

To investigate the effect of inoculation doses on clinical symptoms and mortality of infected mice, 7-day-old ICR mice were infected with CVA19 at the dose of 10^4^, 10^6^, 10^8^, 10^10^ copies/mouse (*n* = 10 per group) via i.g. route, respectively. Our results showed that the body weights of infected mice were significantly lower than that of mock-infected mice (Figure 1(A-a)). CVA19-infected mice began to show symptoms at 3 dpi, such as weight loss, limb weakness, or contracture, but the higher dose groups (10^8^, 60%; 10^10^, 50%) had a higher proportion of these clinical symptoms. At 4 dpi, 1–2 mice in each of the four infected groups began to exhibit severe symptoms, and the mean clinical scores of infected mice were significantly increased compared with control mice (Figure 1(A-b)). Most of the severe infected mice died quickly after becoming severity, but a few of mice with severe illness could last for nearly a week. In our repeated experiments, a few severely infected mice with a low-dose CVA19 challenge could gradually recover from limb paralysis, but it took nearly a month. Mice infected with the doses of 10^8^, 10^10^ copies per mouse started to die at 5 or 6 dpi, and all were dead at 12 and 10 dpi, respectively. With the inoculation doses of 10^4^, 10^6^ copies per mouse, mice began to show dying or death at 7 dpi, and the survival rates were 80% and 30%, respectively (Figure 1(A-c)). Therefore, the onset time points of clinical symptoms and survival rates exhibited a correlation. The infective doses of 10^8^ copies/mouse were chosen for subsequent experiments.

To compare the susceptibility of different age mice, 5-, 7-, and 10-day-old ICR mice were i.g. inoculated with CVA19 (10^8^ copies/mouse), respectively. As shown in Figure 1(B), 5- and 7-day-old mice infected with CVA19 exhibited weight loss (Figure 1(B-a)) and higher clinical scores (Figure 1(B-b)) and all died at 10, 12 dpi, respectively (Figure 1(B-c)). Whereas 10-day-old infected mice developed clinical symptoms at almost 6 dpi and showed lower clinical scores than other infected groups. Subsequently, these mice turned out to be healthy ultimately, and the survival rate was 87.5%. In contrast, control mice exhibited healthy throughout the experiments. However, the maneuverability and repeatability of 5-day-old mice were not as good as 7-day-old mice due to their small size and limited volume of virus inoculation. Figure 1(C) showed hind limb paralysis and weight loss of infected mice at 5 dpi (Figure 1(C-a)), 9 dpi (Figure 1(C-b and c)), and 14 dpi (Figure 1(C-d and C-e)), also Figure 1(C-e) exhibited a severe infected mouse with posterior paralysis and forelimb atrophy, which became healthy at 30 dpi (with later onset, about 9 dpi). The degree of limb involvement ranged from mono-paresis to quadriparesis as the black arrows indicated, leading to a reduced ability to crawl. These results indicate that the CVA19 strain is naturally adapted to mice and induces limb paralysis in neonatal mice with an age- and dose-dependent manner, and 7-day-old mice were more suitable for modelling CVA19 infection.

### Presence and replication of CVA19 in mice tissues

**Figure 2.**
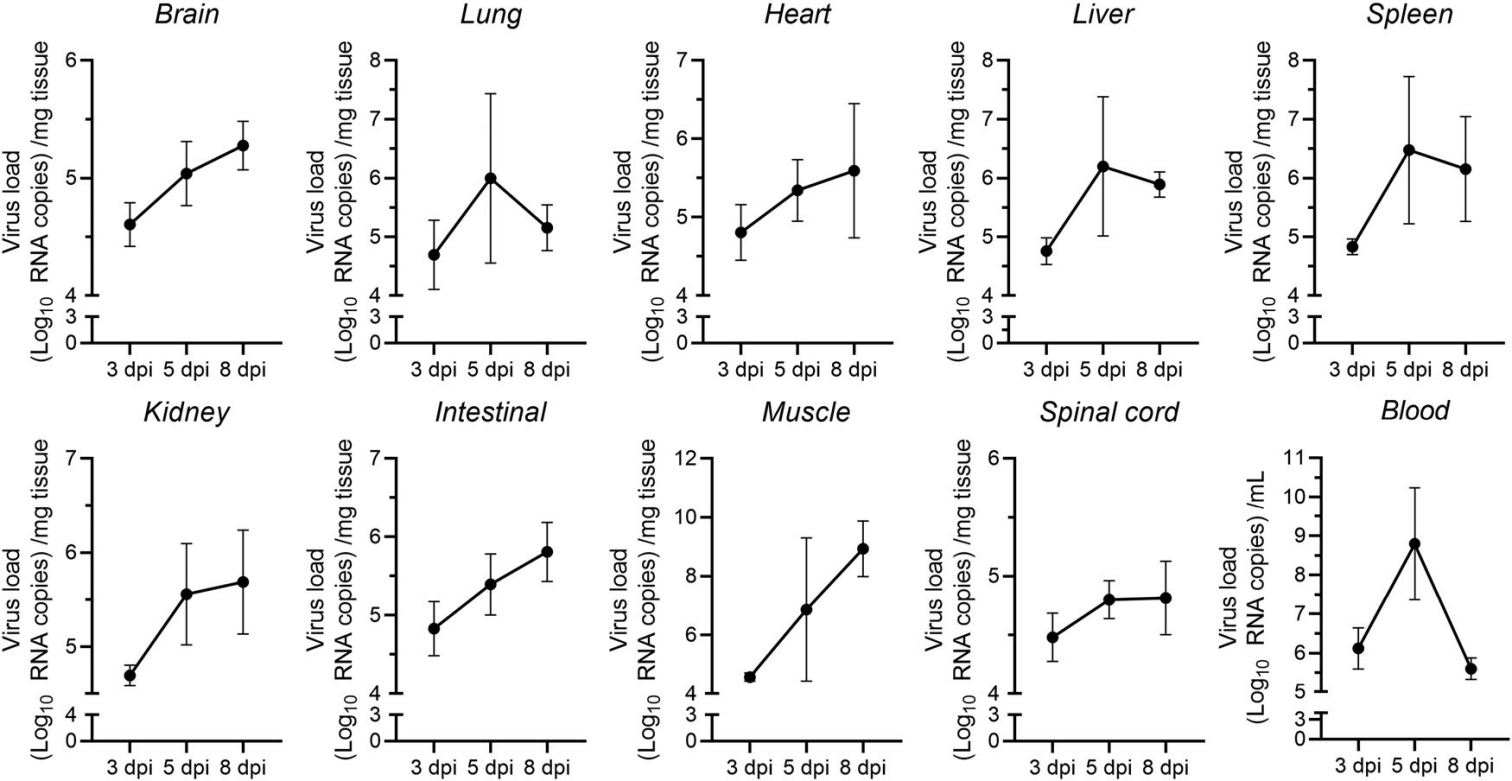
Viral loads in multiple organs. Seven-day-old ICR mice were i.g. inoculated with a lethal dose of CVA19. At 3, 5, and 8 dpi, infected mice (*n* = 4) were euthanized and viral loads were assessed by qRT-PCR in tissue samples. Results represent the mean viral load (Log_10_ (viral RNA copies/mg or mL) tissues or blood) ± SD.

To determine the dynamic mode of viral dissemination after inoculation with CVA19 via i.g. route in 7-day-old mice, the viral loads in brains, lungs, hearts, livers, spleens, intestines, kidneys, muscles, blood and spinal cords of infected mice at 3, 5, and 8 dpi were detected by qPCR. As shown in Figure 2, viral replication was detected in almost all organs or tissues at the early period (3 dpi), especially blood, intestines, hearts, and spleens indicating that these tissues might be the sites of viral replication at the early stage of infection. A higher viral load in the blood implied early viremia. With the progress of infection, the viral loads showed a rapid increase in various organs or tissues from 3 to 5 dpi, suggesting that CVA19 had infected the entire body. The viral loads in the muscles, spleens, livers, and lungs increased more rapidly than those in the other tissues. However, the viral loads in blood, spleens, livers, and lungs did not remain stable at high levels but reduced gradually after reaching a peak at 5 dpi. Moreover, the viral loads in other tissues, in addition to muscle, still increased slowly. In skeletal muscles, the viral loads reached up to almost 10^9^ copies/mg at 8 dpi, 3–5 orders of magnitude greater than those in other tissues or organs, indicating a muscle tropism. Notably, the viral loads in spinal cords and brains were relatively lower at different time points. Apparently, the time course of the viral reproduction practically corresponded to the clinical presentation of the mice.

### CVA19 directly infects brain neurons and further induces neurological disease

**Figure 3.**
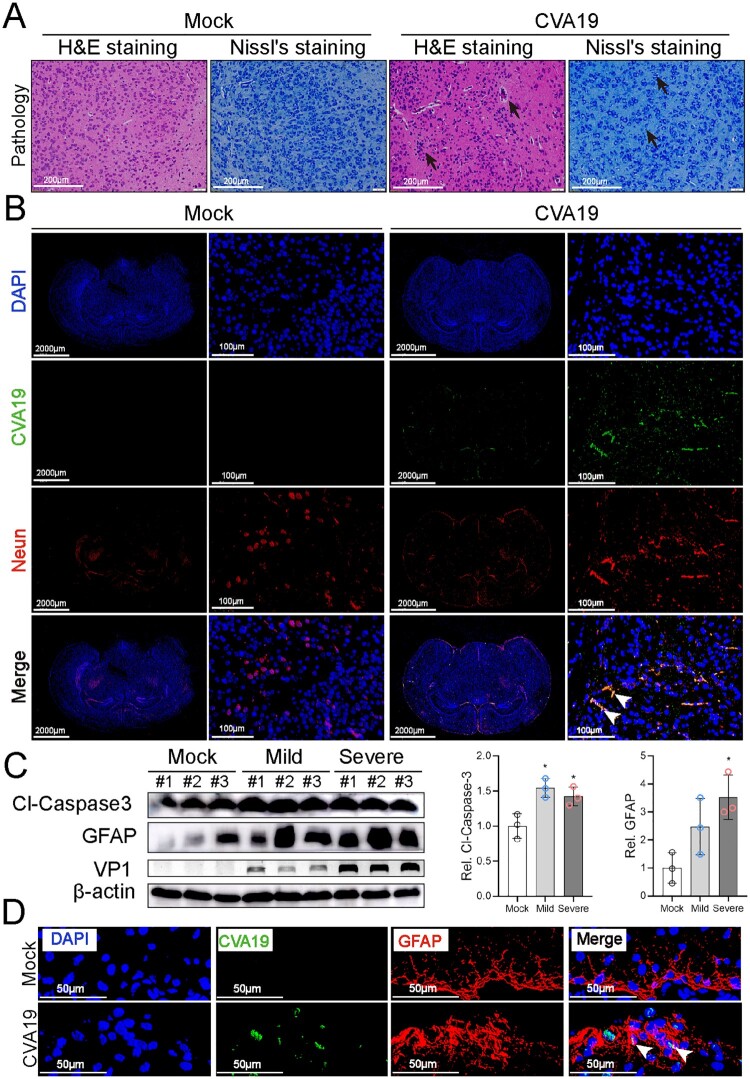
CVA19 directly infects brain neurons and induces neurological pathological damage. H&E staining and Nissl staining (A) were used to evaluate the pathological changes of brains in severe infected mice. The black arrows indicated typical pathological changes. (B) Fluorescence signals representing viral RNA in the neurons (cerebral cortex) were indicated by white arrows utilizing IF and FISH testing. WB analysis (C) was used to detect the expressions of cleaved-Caspase3 and GFAP in brains (*n* = 3 per group). The representing VP1 band was the agarose gel electrophoresis image (917 bp, primer sequence: Forward, GACACACAGTCTAGCGGACC; Reverse, TTGACGGCCTTCTCCATGTC). The protein expression level was expressed as mean ± SD. (D) Fluorescence signals representing viral RNA in the astrocytes (hippocampus) were indicated by white arrows utilizing IF and FISH testing. The variance analysis was conducted by two-tailed Student’s *t*-test on performing the comparison of two groups. Mild or severe group *vs* mock, * *P *< 0.05.

HEVs infection is more frequently associated with severe diseases such as fatal encephalitis. In mock-infected mice, H&E and Nissl’s staining showed that the neurons were distributed in a uniform fashion. In severely infected mice, H&E staining distinctly showed pathologies of possible vasculitis featured varying degrees of perivascular cuffing by inflammatory cells, as well as parenchymal inflammatory cell infiltration with the formation of microglial nodules and focal neuronophagia (black arrows). The morphology of some neurons had a disordered arrangement and the number of Nissl’s bodies was slightly reduced (Figure 3(A)). Next, to determine whether CVA19 could infect brain neurons, we performed immunofluorescence colocalization of viral RNA and NeuN (a specific biomarker of neurons). Fluorescence signals representing viral RNA diffusely distributed in the neurons of infected brains (mainly in cerebral cortex, hippocampus, thalamus) as indicated by the white arrows (cerebral cortex) (Figure 3(B)). We then detected the expression of cleaved-Caspase3 and GFAP in the brains of controls, mild, and severely infected mice, and found that the expression of cleaved-Caspase 3 was increased almost 1.5 folds (both groups of mild and severe). The expression level of GFAP was increased continuously from 2.5 folds in mildly infected mice (without statistical significance) to 3.5 folds in severely infected mice compared with control mice, respectively (Figure 3(C)). The CVA19^+^ GFAP^+^ cells in CVA19-infected brain tissue (mainly in hippocampus) were also detected, indicating CVA19 could also directly infect astrocytes (Figure 3(D)). Taken together, CVA19 directly infects the neurons and astrocytes of mouse brains and further induces neurological disease.

### CVA19 infects motor neurons of spinal cord and further leads to acute flaccid paralysis

**Figure 4.**
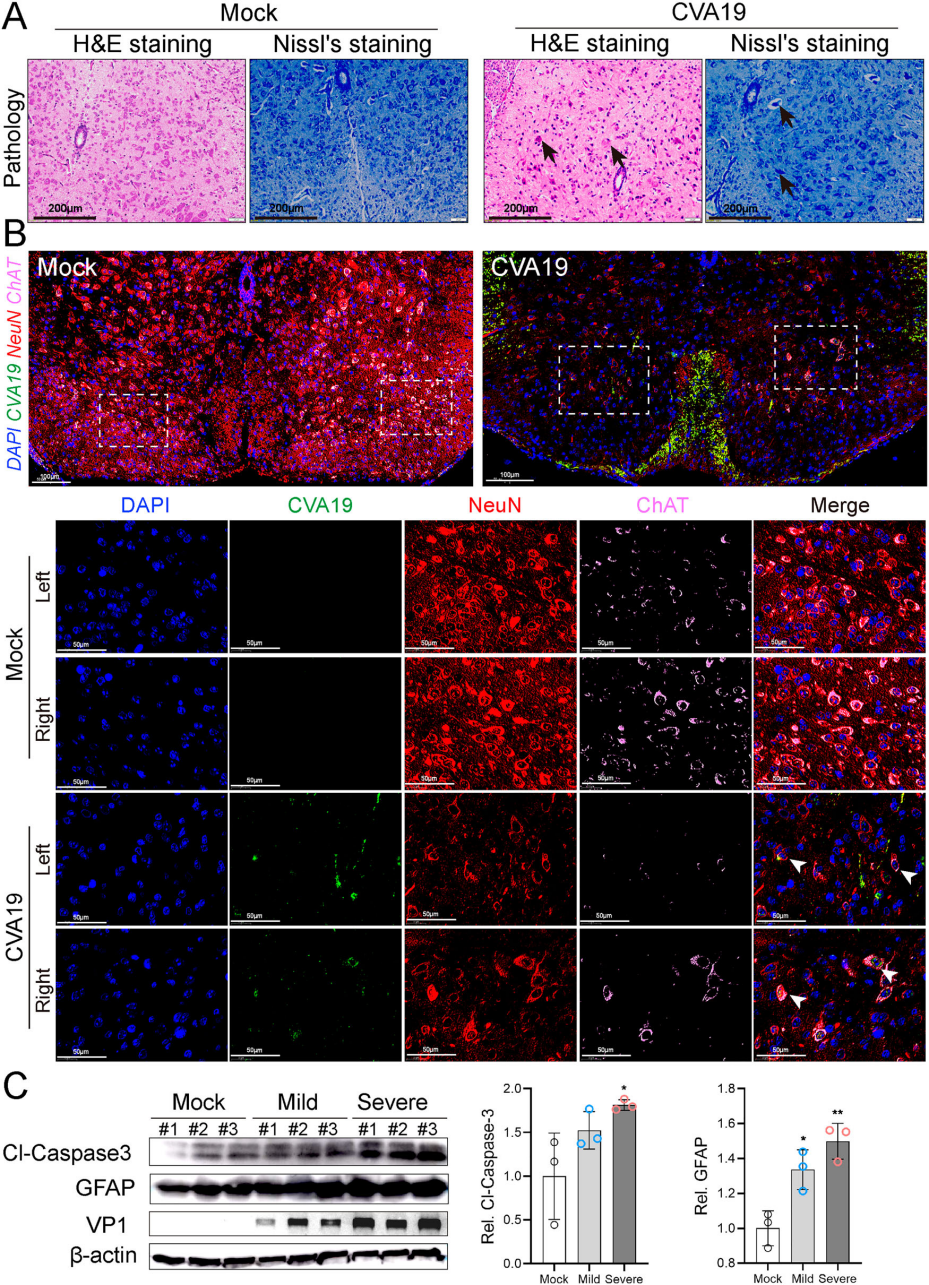
CVA19 infects motor neurons of spinal cord and further leads to acute flaccid paralysis. (A) Histopathological examination of spinal cords from infected severe mice by H&E staining and Nissl’s staining. The black arrows indicated karyopyknotic and hyperchromatic nuclear, reduced or disappeared Nissl bodies, as well as the vacuolization of nuclei. (B) Strong viral RNA signals and apparent loss were detected in spinal motor neurons of both the left and right horns (white arrows) in CVA19-infected mice. The white dashed boxes marked the sites where the images were taken from (anterior horn of the spinal cord). (C) WB analysis was applied to detect the expression of cleaved-Caspase3 and GFAP in the spinal cords (*n* = 3 per group). The representing VP1 band was the agarose gel electrophoresis image (917 bp). The protein expression level was expressed as mean ± SD. The variance analysis was conducted by two-tailed Student’s *t*-test on performing the comparison of two groups. Mild or severe group *vs* mock, * *P *< 0.05, **P < 0.01.

The primary site of injury for HEVs-associated AFP is the anterior horn cell of the spinal cord [24]. Histopathological examination showed loss of Nissl’s bodies, disorderly arrangement, and degeneration of neurons in the spinal cords of infected mice. Further, the vacuolization of nuclei was observed in some neurons (Figure 4(A)). No pathological change was found in mock-infected mice. Anterior horn motor neurons were marked by the colocalization of choline acetyltransferase (ChAT) and NeuN. Viral RNA was signalled by fluorescence in-situ hybridization. As shown in Figure 4(B), a large number of neurons were neatly arranged (red), and motor neurons marked by ChAT (pink) were evenly distributed in the left and right corners of the spinal cords of mock mice, and no viral nucleic acid signal was detected. Fluorescent staining of the spinal cords removed from severe CVA19-infected mice (quadriplegia) exhibited apparent loss of ChAT and NeuN staining, indicating the reduction numbers of motor neurons in the anterior horn of spinal cords corresponding to the affected limb. Importantly, the injury and loss of left motor neurons were more distinct, corresponding to the more severe left limb. At the same time, strong viral RNA signals were detected in spinal motor neurons of both the left and right horns, which represented that CVA19 could infect motor neurons. We also detected the expression of cleaved-Caspase3 and GFAP in the spinal cords of control, mild, and severely infected mice (Figure 4(C)). The expression levels of both cleaved-Caspase3 and GFAP of mild-infected mice were all elevated compared with control mice, but only the change of GFAP had statistical significance. However, the expression levels of the two proteins in the spinal cords of severe infected mice were all significantly increased compared with control mice. Our results suggest that CVA19 infects motor neurons of spinal cord and further leads to acute flaccid paralysis.

### CVA19 leads to ultra-pathological changes in infected mouse spinal cords

**Figure 5.**
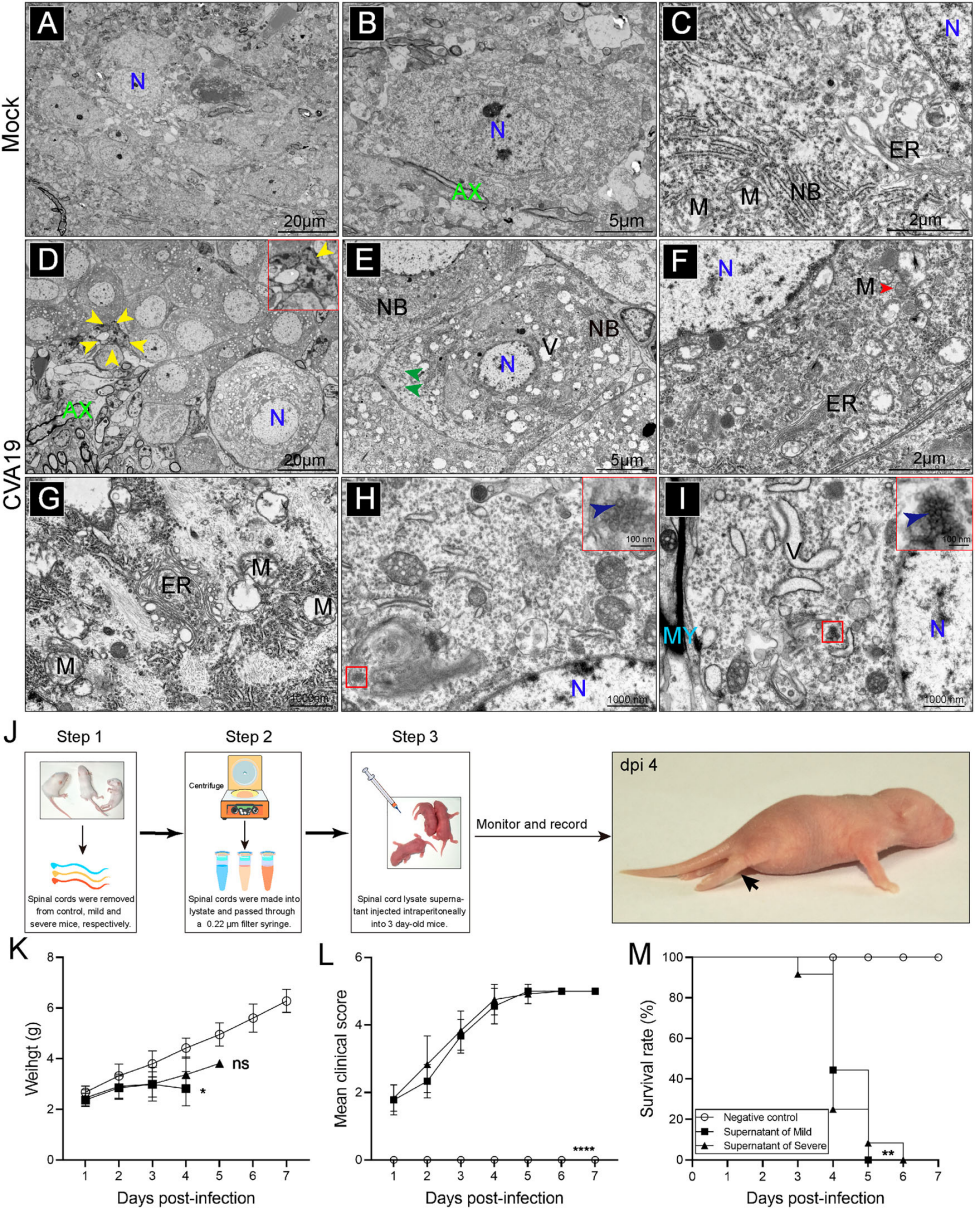
Ultrastructure of spinal cord neurons after CVA19 infection. Inoculation of lysis supernatants of spinal cords from infected mice leads to limb paralysis in 3-day-old mice. (A–C) Ultrastructure of uninfected spinal cord neurons. Intact nuclear (N); Endoplasmic reticulum (ER); Mitochondria (M); Axon (AX); Nissl’s bodies (NB). (D–I) Ultrastructure of infected spinal cord neurons. Pyknotic nucleus and indistinguishable indented nuclear membrane (D, yellow arrows); Disappearance or decrement of Nissl substance (E, green arrows). Relatively dense atrophic mitochondria (F, red arrow). ER with swollen cisternae, dilation and irregular arrangement of mitochondrial cristae (F and G). CVA19 particles in infected neurons (H and I, blue arrows). Vacuoles (V); Myelin sheath (MY). The scale bar has been indicated in the figures. (J) The procedure of the experiment and the production steps of the spinal cord lysate supernatant. The black arrow indicated typical paralysed limbs. The weight (K), clinical manifestation (L), and survival rates (M) were monitored and recorded daily until 7 dpi. The Mantel–Cox Log-rank test was used to compare the survival rates of mice in different groups. The variance analysis was conducted by a two-tailed Student’s *t-test* on performing the comparison of two groups. Data represent the mean ± SD. Different groups *vs* negative control, *P < 0.05, **P < 0.01, ****P < 0.0001; ns, non-significant.

We next used electron microscopy to examine ultrastructural localization of viral particles, as well as the fine-structural changes of spinal cord neurons at the later stage of infection. As shown in the mock-infected mice (Figure 5(A–C)), neurons ranging in diameter from 25 to 60 μm with normal electron density and morphology were neatly scattered throughout the ganglia. In CVA19-infected mice, the number of various sizes vacuoles (V), distributed throughout the cytoplasm of infected neurons, were significantly increased (Figure 5(D and E)). We observed the pyknotic nucleus and indented nuclear membrane (Figure 5(D)). Some glial cells containing ingested material in the cytoplasm were observed around degenerating neurons, suggesting high phagocytic activity (Figure 5(D)). Along with the vacuolization of the neuronal cytoplasm, there was an accompanying disappearance or decrement of Nissl’s substance (Figure 5(E)). ER with swollen cisternae was very sparse, and the number of free ribosomes in the cytoplasm appeared to be elevated. Dilation and irregular arrangement of mitochondrial cristae, as well as diminished density of the mitochondrial matrix were identified (Figure 5(F and G)). At the same time, we also found relatively dense atrophic mitochondria (Figure 5(F)). Electron micrographs of higher magnification showed CVA19 particles presented a constant size and a consistent substructure in infected neurons, ranging in diameter from 25 to 40 nm (Figure 5(H and I)). A few of progeny virions were enclosed individually within small vesicles. In conclusion, CVA19 directly infects neurons of the spinal cord resulting in neurological disease.

### Inoculation of lysis supernatants of spinal cords from infected mice leads to limb paralysis in suckling mice

To determine whether CVA19 was the direct cause of the paralytic disease in mice by fulfilling Koch’s postulates to some extent, lysis supernatants of spinal cords from mild and severely infected mice were administrated by i.p. injection based on 3-day-old suckling mice. The procedure of the experiment and the production steps of lysis supernatants of spinal cords were shown in Figure 5(J). Almost no weight gain was observed in mice injected with lysis supernatants from infected mice compared with control mice (Figure 5(K)), but the difference between the mice injected with lysis supernatants from mild-infected mice and negative controls had a statistically significance. After mice were inoculated with lysis supernatants of spinal cords from infected mice, the infected mice appeared reduced mobility, limb weakness, and paralysis with gradually increased mean clinical scores (Figure 5(L)) and all died within 5 dpi (mild supernatant) and 6 dpi (severe supernatant), respectively (Figure 5(M)). No obvious abnormalities were seen in control mice. Figure 5(J) showed a picture of a typically paralysed suckling mice and the black arrow indicated paralysed hind limbs. Together, the virus contained in lysis supernatants of spinal cords from infected mice leads to limb paralysis in newborn mice, suggesting CVA19 is the direct cause of paralysis to a certain extent.

### CVA19 leads to small bowel inflammatory injury and diarrhoea in mice

**Figure 6.**
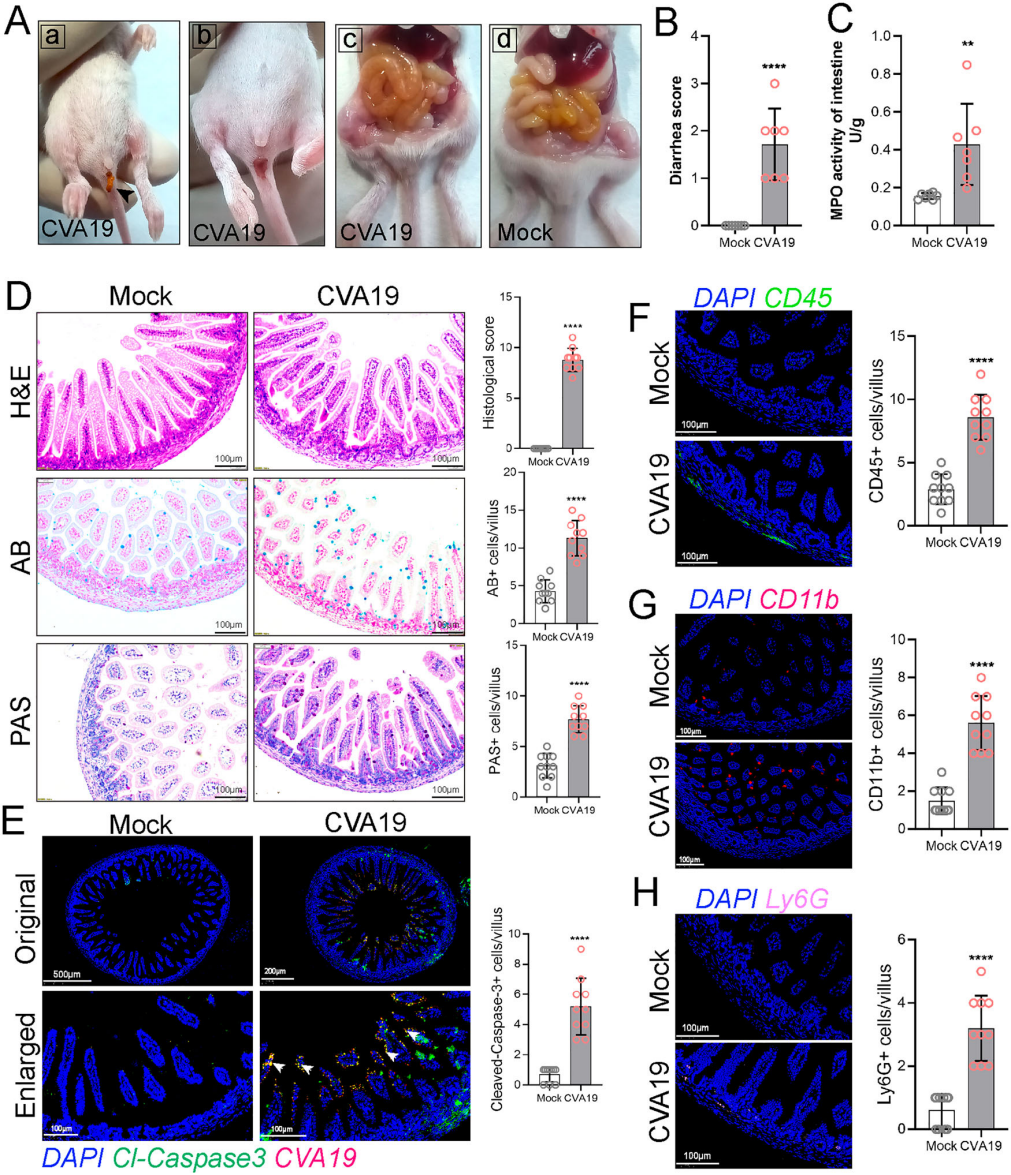
CVA19 leads to small bowel inflammatory injury and diarrhoea in mice. Diarrhoea symptoms in infected mice (A-a) and the black arrow indicate soft yellow faeces. (A-b) Perianal staining of the coat. Macroscopic observations of the intestine of representative mice from CVA19-infected mice (A-c) and mock mice (A-d) at 6 dpi. (B) The severity of diarrhoea (*n* = 7 per group) was scored at 6 dpi (0, normal stool or absent; 1, slightly wet and soft stool; 2, wet and unformed stool with moderate perianal staining; 3, watery stool with severe perianal staining). (C) The MPO activity of small intestine (*n* = 7 per group). (D) The pathology of small intestine was assayed at 10 dpi according to H&E, PAS, and AB staining. (E) The colocalization of CVA19 viral RNA and cleaved-caspase3 (white arrows). IF was performed to localize leukocytes (F), monocytes (G), and neutrophils (H). Data were expressed as mean ± SD and analysed by Student’s *t* test. CVA19 group *vs* mock groups, **P < 0.01, ****P < 0.0001.

Previous studies reported diarrhoea in CVA19 infections [7,8]. To explore whether intestinal injury was also a feature in CVA19 mouse model, we investigated the effect of CVA19 infection on intestinal mucositis. Diarrhoea was defined as unformed stool upon daily examination and sampling. As shown in the photographs in Figure 6(A-a and A-b), the faeces were yellow, soft or unformed as noted by black arrow and the perianal coat were stained. At 6 dpi, serious gaseous distention, capillary congestion in mesenteries, and large amounts of yellow foamy liquid in the intestine were observed, especially in the jejunum (Figure 6(A-c)) compared with mock mice (Figure 6(A-d)), and the mean diarrhoea scores achieved 1.71 (Figure 6(B)). Additionally, the MPO activity of the intestine was significantly elevated in the CVA19-infected mice at 6 dpi (Figure 6(C)), further indicating intestinal injury. As shown in Figure 6(D), normal intestinal histopathology was observed in small intestine sections of mock mice. However, the small intestine of CVA19-infected mice showed shortened villi, inflammatory cell infiltration, and complete destruction of the tissue architecture, resulting in extensive damage with higher histopathologic scores. Meanwhile, the goblet cell number secreting acid mucins (AB positive cells) and neutral mucins (PAS positive cells) in the small intestine were increased in CVA19-infected mice, exhibiting increased mucus secretion. We next examined the distribution of CVA19 in the small intestine, and found that CVA19 viral RNA and cleaved-Caspase 3 co-localized in small intestinal villi cells (as indicated by the white arrows), and the expression level of cleaved-Caspase 3 was higher in the infected mice, indicating that viral infection led to apoptosis in small intestine (Figure 6(E)). We then performed IF to localize and quantify diarrhoea-related immune cells in small intestinal tissue, including leukocyte (CD45^+^ cells), monocytes (CD11b^+^ cells), and neutrophils (Ly6G^+^ cells). Our results showed that a large number of leukocytes were infiltrated in the small intestinal villi and intestinal wall tissue (Figure 6(F)). Whereas, monocytes were mainly scattered in the tip of villi of the small intestinal (Figure 6(G)), and neutrophils were mainly concentrated in the small intestinal wall tissue (Figure 6(H)). The number of these immune cells in CVA19-infected mice was significantly increased compared with mock mice. Taken together, these data indicate that CVA19 infection leads to small bowel inflammatory injury and diarrhoea.

### CVA19 infection induces a systemic inflammatory response

**Figure 7.**
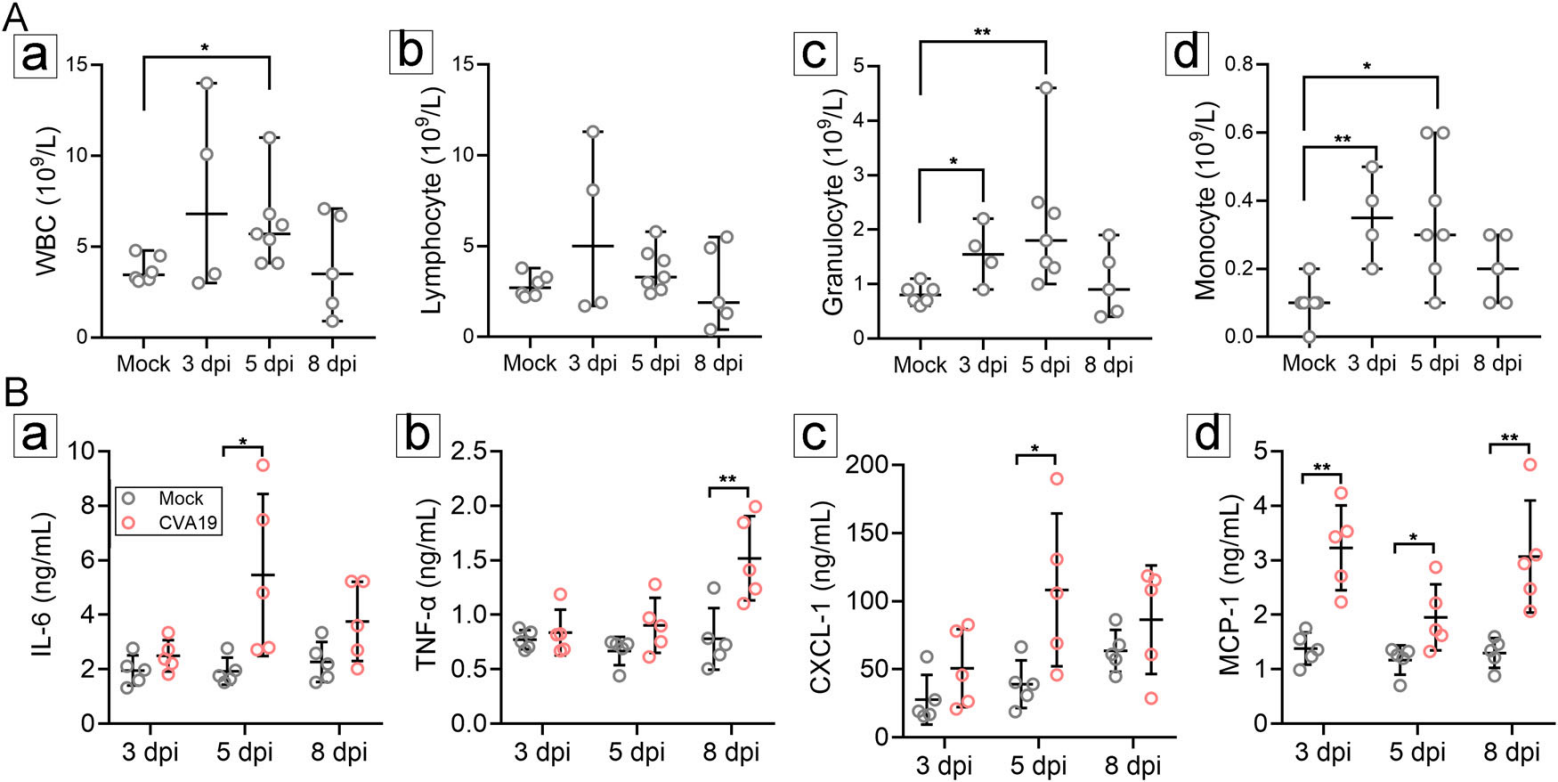
CVA19 infection induces a systemic inflammatory response. The numbers of white blood cell (WBC) (A-a), lymphocytes (A-b) as well as granulocyte (A-c) and monocyte (A-d) of CVA19 infection group were detected at 3, 5, and 8 dpi (*n* = 4–7 per group). The concentrations of IL-6 (B-a), TNF-α (B-b) CXCL-1 (B-c), and MCP-1 (B-d) in serum were measured by ELISA. The results were expressed as the mean ± SD or median with range. The difference analysis was conducted by two-tailed Student’s *t-test* or Mann-Whiteny test. Different groups *vs* mock group, *P < 0.05, **P < 0.01.

The imbalance of immune homeostasis is one of the main causes of pathological damage. Therefore, we detected immune-cell number and cytokines in the peripheral blood from mock- and CVA19-infected mice at 3, 5, 8 dpi, respectively. The number of white blood cells (WBC) (Figure 7(A-a)), as well as granulocyte (Figure 7(A-c)) and monocyte (Figure 7(A-d)) of CVA19-infected mice was significantly elevated at 5 dpi compared with mock mice. Moreover, the number of granulocyte and monocyte in CVA19-infected mice was significantly increased at the early stage (3 dpi) (Figure 7(A-c and A-d)). The number of lymphocytes was increased slightly at 3 and 5 dpi, but the difference had no statistical significance (Figure 7(A-b)). Abnormal elevated serum proinflammatory cytokines of IL-6, TNF-α, CXCL-1, and MCP-1, are biomarkers of severe illness. Hence, we determined the concentrations of serum cytokines after CVA19 infection. Specifically, serum proinflammatory cytokines, such as IL-6 (Figure 7(B-a)) and TNF-α (Figure 7(B-b)) were significantly increased at 5 and 8 dpi, respectively. Moreover, the concentrations of CXCL-1 (Figure 7(B-c)), MCP-1 (Figure 7(B-d)) were also significantly elevated, which recruited neutrophils and monocytes to the site of infection. Taken together, our results indicate that CVA19 induces a systemic inflammatory response.

### CVA19 antisera containing neutralizing antibodies prevents severe illness caused by CVA19

**Figure 8.**
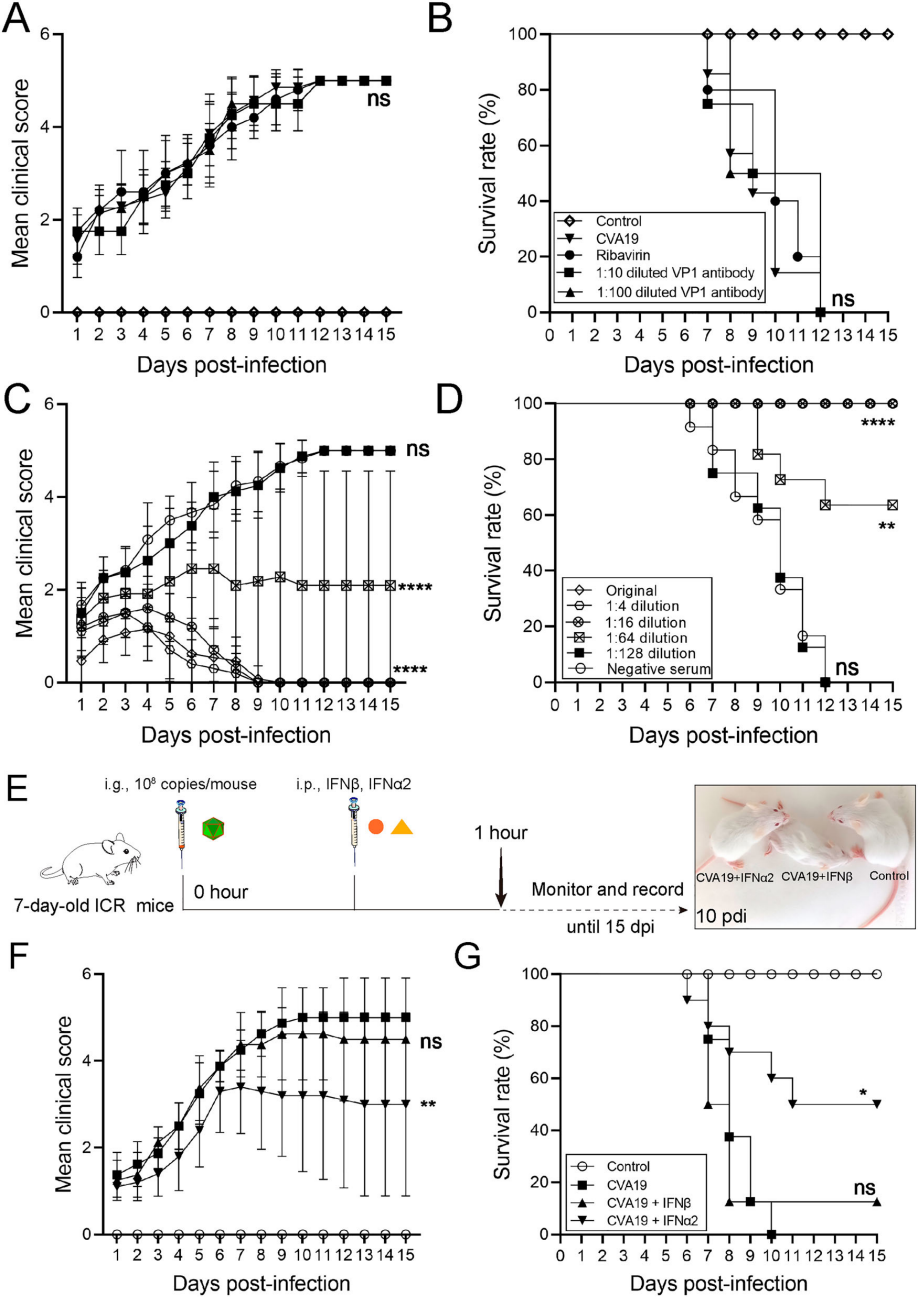
The protective efficacy of ribavirin, CVA19 VP1 mAb and CVA19 antisera, type I IFNs. Groups of 7-day-old neonatal ICR mice (*n* = 8–10 per group) were inoculated with CVA19 via the i.g. route. Within 1 h after infection, mice were given corresponding treatments as mentioned above. The clinical manifestations (A and C) and mortality (B and D) were monitored and recorded daily until 15 dpi. (E) The experimental procedure. The clinical symptoms (F) and mortality rate (G) were monitored and recorded daily until 15 dpi. The Mantel-Cox Log-rank test was used to compare the survival rates of mice in different groups. The difference analysis was conducted by two-tailed Student’s *t-test*. Data represent the mean results ± SD. Different groups *vs* CVA19 group, *P < 0.05, **P < 0.01, ****P < 0.0001; ns, non-significant.

For protection studies, a mouse model of the lethal form was treated with ribavirin and CVA19 VP1 mAb at 1 h post challenge (Figure 8(A)). However, there was no decrease in the mean clinical scores (Figure 8(A)) and died within 7–12 dpi (Figure 8(B). Next, to evaluate the protective effect of the CVA19 antisera on the mice by passive immunization, 50 μL of original, 4-fold serially diluted antisera (1:4–1:128) or the negative serum was i.p. injected into 7-day-old mice after inoculating with a lethal dose of CVA19. The results showed that antisera could significantly improve survival rates and alleviate clinical symptoms post infection. The mean clinical scores for different dilutions were reduced with increasing quantities of antisera (Figure 8(C)). A significant dose–response effect was observed between the concentrations of antisera and the survival rates (Figure 8(D)). Taken together, the above results suggested CVA19 antisera containing neutralizing antibodies prevented severe illness caused by CVA19, not ribavirin and CVA19 VP1 mAb.

### Recombinant IFN-α2 therapy reduces severe illness induced by CVA19

As shown in Figure 8(E), we characterized the antiviral effects of type I IFNs in CVA19-infected mice. After the challenge, the infected mice without treatment all died between 7 and 10 dpi. The mice treated with IFN-β showed no signs of recovery versus CVA19 groups (Figure 8(F and G)). In contrast, some mice with IFN-α2 therapy suffered from mild symptoms, and gradually recovered from 7 dpi (Figure 8(F)) and the ultimate survival rate was 50% (Figure 8(G)). Taken together, the recombinant IFN-α2 therapy reduces severe illness induced by CVA19.

## Discussion

Although, the majority of Enterovirus (EV) infections are asymptomatic and self-limited, a variety of infections display a wide range of severe illnesses, including non-specific febrile illness, HFMD, encephalitis, myocarditis, AFP, gastroenteritis, respiratory diseases, and so on [25]. In recent years, several non-polio EVs have emerged as serious public health concerns. We obtained a CVA19 strain in a stool sample from a child with HFMD, which was reported in our previous study [11]. As a rare serotype of HEV-C, CVA19 is associated with gastroenteritis [7,8], aseptic meningitis [10], AFP [26] as well as HFMD [11]. Although EVs infections can result in a wide range of clinical diseases, HFMD is mainly caused by HEV-A and HEV-B. HEV-C were detected almost exclusively in gastrointestinal (GI) samples, and never reported in HFMD case, which highlighted the emerging threat of the virus to human health. The detection of CVA19 in HFMD patients implies the expansion of the symptom spectrum and the enhancement of pathogenicity. Similarly, it enriches the pathogen spectrums of HFMD, suggesting that CVA19 has evolved into a life-threatening neurotropic virus. However, the current body of knowledge on CVA19 infection is still very limited, particularly the pathogenesis of CNS and digestive system. Our study provides a scientific animal model for further exploring pathogenic mechanism of CVA19, and developing antiviral drugs, vaccines. It is of great significance for the prevention and control of CVA19-related diseases.

Unlike most of the EVs, coxsackieviruses A are generally difficult-to-cultivate in *vitro*, which impedes their propagation and isolation from clinical material. Generally, the efficiency of EVs infection in mouse cells is extremely low because of the difference of receptors. However, a previous study showed that newborn mice were sensitive to difficult-to-cultivate CVA22, and the virus productively multiply in the primary culture of mouse skeletal muscle [27]. For a long time, CVA19 is also refractory to propagation in any cell system tested, but can proliferate in suckling mice [28]. The CVA19 strain used in the present study was also cultivated in new born mice, with the development of a flaccid paralysis being the endpoint for the identification of infection. Some attachment receptors may be a major selection factor for cell culture adaptation, which needs further studies to deeply investigate.

In the present study, CVA19-infected mice displayed typical neurological symptoms, suggesting CVA19 could spread from the GI tract to the CNS and finally induce neurological disease. This model simulates the main symptoms of human infection. Unfortunately, the infected animals did not develop a papulovesicular rash on extremities and oral ulcers, which were clinical features of HFMD. The virulence of CVA19 for mice showed obvious dose- and age-dependent manners. Both viral (polymorphisms in nucleotides and viral loads) and host factors (susceptibility and immunity) are thought to be associated with the severity of enterovirus-related disease. A recent study has shown that HFMD severity is positively correlated with viral loads in throat swabs [29]. Moreover, our study showed mice over 10-day-old were not susceptible to CVA19. The lethality of CVA19 strain was relatively mild compared with the JLS10 CVA6 strain (Accession number: OM179765), which could infect older suckling mice (10-day-old) with a shorter course of disease (all died within 3 days) in our previous study [30]. EV infections occur most frequently in children and the age-dependent susceptibility to EVs has been subsequently confirmed in numerous mouse models [31]. It is widely acknowledged that some kind of age-dependent manner of the intestinal immune system maturation and epithelial tolerance restrict paediatric infection [32]. Also, some unknown EV receptors are expressed in muscle or nerve only transiently at the neonatal age, but rapidly turned off at an older age during mouse development. However, what kind of viral or host factors contribute to the age dependency remains to be investigated.

The spread and amplification of virus within the host system is of great importance for the understanding of disease. Our study revealed that CVA19 replication occurred in various tissues and organs. Obviously, virus replication was first observed in the intestine, which was the site of primary proliferation because of the i.g. route. Relatively high viral copies (almost 10^5^ copies/mg) could be detected in the intestinal at the early stage (3 dpi), followed by heart and spleen. The virus was detected in the spleen relatively early with a very high virus titre, indicating that the organ might serve as an amplification place. In the skeletal muscles, the viral loads at the early stage were not very high, but reached up to almost 10^9^ copies/mg at 8 dpi, 3–5 orders of magnitude greater than those in the other tissues and organs resulting in massive necrosis in limb muscles. The skeletal muscles were also a target of CVA19 infection and a major viral replication site for viral replication. Unfortunately, the strong muscle tissue tropism of CVA19 infection in neonatal mice is significantly different from that in human infection. It is possible that the skeletal muscle serves as an intermediate reservoir of CVA19 during the spreading from gut to other organs or tissues. It is very common to observe muscle infection with enterovirus in mouse models, but myositis in humans has not been reported [33]. Notably, the viral loads in the spinal cord and brain were detected, despite with relatively lower at different infection points, indicating CVA19 successfully entered the CNS. Hence, it is difficult to sort out definitively whether limb paralysis is caused primarily by muscle or CNS injury. Most likely, both muscle and CNS infections contribute to limb paralysis. At present, it remains unclear how CVA19 spreads from gut to other tissues via an oral route. RNA-seq analyses showed that there was massive up-regulation of the genes related to neuronal communication in the gut, suggesting that EVs might enter the CNS via the network of neurons lining guts using active retrograde axonal transport [34]. Even though researchers demonstrated that retrograde axonal transport in neuron cells, but not haematogenous transport, might be the major transmission route of EV71 in mice [35]. The possibility cannot be excluded that CVA19 somehow passes through the blood–brain barrier at a lower efficiency in infected mice. Taken together, we propose that CVA19 may initially infect some cells early and continuous replication in the intestines. Next, the virus is passed into the blood or lymphoid circulation, and further proliferates in some tissues such as muscle and spleen. Finally, the virus invades the CNS via the blood–brain barrier, or retrograde axonal transport along peripheral or cranial nerves [36].

The neurological manifestation (limb paralysis, similar to the symptoms of AFP) and the CNS lesions of CVA19-infected mice reflect the neurotropism and neurovirulence of the virus. The prototype strain of CVA19 was isolated in Japan in 1952, which was associated with Guillain-Barré syndrome (GBS) [37]. GBS is a common cause of AFP, characterized by symmetrical weakness of the limbs, and hyporeflexia or areflexia. Symptoms of a respiratory and GI tract infection before the onset of GBS were reported in two-thirds of patients [38]. In the present study, a positive signal of CVA19 RNA was detected mainly in brain and spinal cord neuronal cell bodies in infected mice, which was consistent with the previous quantitative test, confirming the predilection of CVA19 for neurons. Furthermore, the infectious virus was detected in the spinal cords of the infected animals to fill Koch’s postulates to some extent [23]. Our results showed infectious viral particles contained in the infected spinal cord could lead to paralysis after infection again. This finding supports that the apparent limb paralysis is caused by CVA19 infection, similar to of EVD68-induced encephalomyelitis in mice [39]. In addition, by H&E and Nissl’s staining, perivascular cuffing, glial nodules, and loss of Nissl bodies were observed in the spinal cords as well as the brain. The involved neurons showed apoptosis, degeneration, and necrosis with neuronophagia and inflammatory infiltration. TEM images of the spinal cord removed from a severe mouse with limb paralysis confirmed the presence of apoptotic neurons, and we also observed ∼30 nm particles morphologically consistent with EVs. The number of vacuoles of various sizes, distributed throughout the cytoplasm of infected neurons, was significantly increased, which might be involved with the viral proliferation [40]. It was further observed that the disappearance or decrement of Nissl’s substance, elevated free ribosomes in the cytoplasm, sparse, and swollen ER, dilation and irregular arrangement of mitochondrial cristae, which was a very basic similarity in the ultrastructural pathology changes of CNS injury caused by other EVs [41,42]. The neuron-tropism of CVA19 supports previous findings in fatal cases of EV71-infected children [43]. Fluorescent staining of spinal cord removed from severe CVA19-infected mice (quadriplegia) exhibited the reduction of motor neuron populations in the anterior horn of the spinal cord corresponding to the affected limb. Additionally, the neurons depletion was very pronounced in the CVA19-infected spinal cord, which was inevitable resulting from a viral infection, immune damage, and other reasons [30,44]. In the mouse model of AFP based on EVD68, the virus infects motor neurons and results in motor neuron death [39]. The inflammatory infiltration involved primarily the grey matter with perivascular lymphocytic cuffing and neuronophagia [43]. MRI reports also showed that the lesion mainly involved the grey matter of the spinal cord in AFP cases [45], which was similar to our results. Furthermore, as the major producer of IFN within the CNS, the activation of astrocytes has also been proposed to be part of the neuropathology, and is thought to participate in development of paralysis caused by EV71 infection [46]. We demonstrated astrocyte activation and infection according to the higher expression of GFAP and colocalization with viral nucleic acid signals in severe mice. The activation of astrocytes was also detected in CVA6 and EV71-infected brain [30,47], suggesting that astrocytes might play a key role in the HFMD-related neurogenic pathogenesis. Overall, CVA19 directly infects the CNS to cause neuronal cell injury, resulting in typical AFP.

As mentioned above, CVA19 infection generally causes diarrhoea clinically [7]. Despite some EV strains are capable of replicating and inducing diarrhoea in the pups, the clinical signs have rarely been reported in HFMD-related models in infected mice [2]. In our present study, we successfully simulated diarrhoea of human infection and the faeces exhibited yellow, soft, or unformed. Further studies provided sufficient evidences of small intestinal injury leading to diarrhoea, such as the colonization and proliferation of CVA19 in the intestinal tract, shortened intestinal villi, increased the number of secretory cells and apoptotic small intestinal cells, and inflammatory infiltration, which was similar with the findings of other enterovirus-associated models [13]. The GI tract is covered by epithelium comprising various cell types, including absorptive columnar epithelial cells, goblet cells, which may be permissive to CVA19 infection. The intestinal mucosal immune system has a complete immune response and immune regulation mechanism, and viral infections may disrupt intestinal immune balance between the pathogen elimination and tissue protection [48]. After CVA19 infection, a large number of inflammatory cells including monocytes and neutrophils were observed in the submucosa of the small intestines. Notable necrotic enteritis with a large number of inflammatory cells (macrophages, lymphocytes, NK cells, neutrophils, and plasma cells) infiltrating the intestinal tissues throughout the lamina propria and submucosa of the intestines was also observed in fatal cases of EV71-related HFMD [49].

Cytokines and chemokines play important roles in disease pathogenesis by regulating inflammatory responses. The CNS dysfunction is due to direct viral invasion or secondary immune-mediated response. A mild to moderate elevation of white blood cell count in cerebral spinal fluid (CSF) is identified in almost all patients with EVD68-related AFP [50]. Many cytokines were expressed at higher levels in the CSF and serum of AFP patients [51]. However, CSF of mouse is difficult to obtain, and we only examined inflammatory cells and cytokines in the blood to evaluate the immune status. The results showed much higher levels of cytokines and excessive immune cells in the blood of CVA19-infected mice compared with corresponding control mice. These changes were usually occurred at the early or/and middle stage of disease, but some factors tended to be normal at the late stage, suggesting that some cytokines or immune cells were the major factors in early inflammatory responses and others might play important roles in the process of severe immunopathological injury [52]. The level of IL-6 increased in the early stage and then gradually decreased in CVA6 infection mice described in our previous study [30], while it increased in the middle stage and decreased in the late stage in current study. Different severity of EV71-associated HFMD patients exhibited respective special inflammation cytokine profiles [53]. Several cytokines or chemokines such as IL-6, TNF-α, IL-8, and MCP-1 have been reported to be associated with severe cases with EVs-related neurological symptoms by numerous studies [51,54].

Ribavirin is an antiviral nucleoside analog that is mutagenic to several RNA viruses, which is widely used in clinical treatment. Moreover, ribavirin has been proved to reduce the mortality, morbidity, and subsequent paralysis sequelae in infected mice by decreasing viral loads in tissues [20,55]. In the present study, the antiviral activity of ribavirin against CVA19 infection was completely absent, as well as against CVA6 infection introduced in our previous study [30], which might be caused by the resistance to ribavirin [56]. Immunization is believed to be the most effective tool to control the EVs epidemic. Capsid proteins especially VP1 with the presence of major B- and T-cell epitopes are the most antigenic proteins, which can efficiently prevent the infection of EVs [57], but passive immunization with CVA19 VP1 mAb was also ineffective in this study. Nevertheless, higher concentrations of the CVA19 antisera provided 100% protection (fatal infection), indicating that importance neutralizing antibodies. Previous studies including our own reported similar results [33,39]. Although the type I IFN response elicited upon virus infection is critical to establishing host antiviral innate immunity, EVs have evolved multiple strategies to evade or subvert the host immunity to ensure their survival [58]. Studies have showed the augments of IFN-α/β production can inhibit viral propagation and improves survival in the mouse models [21,59]. In the present study, IFN-α2 had higher antiviral activities against CVA19 *in vivo* at the early stage of infection, which significantly reduced disease severity induced by CVA19. Likewise, the administration of IFN-α2 attenuates EV71-induced nerve damage [60]. Conceivably, there are deviations of IFNs against different serotypes EVs, possibly reflecting the protective differences of serotype-specific and different dosing time.

## Conclusion

In conclusion, this study suggests that a natural mouse-adapted CVA19 strain leads to diarrhoea and encephalomyelitis in a mouse model via oral infection, which provides a useful tool for studying CVA19 pathogenesis and evaluating the efficacy of vaccines and antivirals.
